# Efficacy of embolo/sclerotherapy in the treatment of high-output cardiac failure caused by peripheral arteriovenous malformations

**DOI:** 10.3389/fcvm.2025.1592077

**Published:** 2025-09-01

**Authors:** Xixi Guo, Qiangqiang Nie, Kai Zheng, Bin Ni, Yuguang Yang, Zhidong Ye, Peng Liu, Xueqiang Fan

**Affiliations:** ^1^ Institute of Clinical Medical Sciences, China-Japan Friendship Hospital, Beijing, China; ^2^ Department of Cardiovascular Surgery, China-Japan Friendship Hospital, Beijing, China; ^3^ Institute of Clinical Medical Sciences, China-Japan Friendship Hospital, Chinese Academy of Medical Sciences & Peking Union Medical College, Beijing, China

**Keywords:** peripheral arteriovenous malformations, high-output cardiac failure, embolo/sclerotherapy, devascularization, pressure of venous drainage

## Abstract

**Purpose:**

This study aimed to evaluate the effectiveness and outcomes of embolo/sclerotherapy in treating Schobinger Stage IV peripheral arteriovenous malformations (pAVMs) associated with high-output cardiac failure (HOCF).

**Methods:**

Between January 2017 and December 2024, 12 patients with Schobinger Stage IV pAVMs and associated HOCF were treated with embolization using coils and sclerosing agents, including bleomycin polidocanol foam (BPF) and anhydrous ethanol. Procedural outcomes, complications, devascularization and improvements in cardiac function were evaluated during follow-up.

**Results:**

A total of 24 embolo/sclerotherapy sessions were performed on the 12 patients. Complete or over 80% devascularization of the vascular malformations was achieved in 9 patients. Echocardiographic follow-up revealed significant improvements in cardiac ejection fraction and ventricular dilatation. Additionally, 8 patients showed improvement in heart valve regurgitation. For all patients, the symptom of dyspnea disappeared after embolization and no serious complications occurred. LVEF improvement showed a significant positive correlation with a decrease in venous drainage pressure of the nidus after surgery (*P* < 0.001) in 11 patients, while one patient was excluded due to an increase in venous drainage post-surgery.

**Conclusion:**

This study provides evidence that embolo/sclerotherapy effectively treats pAVMs with associated HOCF by reducing abnormal blood flow and significantly improving symptoms. The results also reveal a linear relationship between the decrease in venous drainage pressure and improvement in LVEF in patients with HOCF caused by pAVMs after embolo/sclerotherapy.

## Introduction

Arteriovenous malformations (AVMs) are congenital vascular abnormalities caused by a birth defect, resulting in direct connections between primitive reticular networks of dysplastic vessels which referred to “nidus” (1). As aggressive congenital high-flow vascular lesions, AVMs are characterized by shunting of high velocity, low resistance flow from the arterial vasculature into the venous system (2), necessitating early intervention. Delayed treatment can lead to chronic vascular “steal phenomenon”, this condition can result in ventricular remodeling and myocardial dysfunction, and eventually progress to high-output cardiac failure (HOCF) (3, 4). AVMs are prone to complications such as pain, pulsations, swelling, and ulcerations. Clinical staging is typically based on the Schobinger classification, with Stage IV indicating systemic symptoms, including cardiac failure. Schobinger Stage IV AVMs usually appear in early adulthood, with symptoms often developing between the ages of 10 and 40, according to the Mayo Clinic and the Cleveland Clinic. While some individuals may have symptoms present at birth or develop them later in childhood, the peak incidence of symptomatic AVMs, including those classified as Schobinger Stage IV, generally occurs within this early adulthood range.

In HOCF, the predominant phenotype is characterized by enlarged cardiac chambers, typically caused by arteriovenous shunts (5). HOCF can cause further morbidity and mortality due to its impact on various complications, including structural heart changes, pulmonary hypertension, and dysfunction of other organs. Effective treatment to reduce the cardiac burden can significantly improve patients' quality of life and survival rates. Conventional heart failure medications offer limited symptomatic relief, whereas treatment aimed at correcting the abnormal arteriovenous shunting results in substantial improvement in heart failure symptoms.

The primary goal in treating pAVMs associated with HOCF is to unload the heart, thereby aiding recovery from cardiac volume overload. Embolization and sclerotherapy have proven clinically effective in managing these patients. Embolization is considered the main endovascular therapy and typically involves the use of various coils. Sclerotherapy, on the other hand, is a widely used, low-risk, and highly successful option for treating malformation. Our previous research has demonstrated the effectiveness of bleomycin-polidocanol foam (BPF) as a sclerosing agent in the treatment of AVMs (6). Polidocanol helps alleviate pain and enhances the ability to better fill the lesion area in foam form (7). Bleomycin further strengthens the sclerosing effect (8). When combined with anhydrous ethanol, which completely destroys vascular endothelial cells and blocks vascular reperfusion, BPF improves treatment outcomes and reduces recurrence rates (2, 9). Therefore, BPF combined with anhydrous ethanol was chosen as the sclerosing agent for this study.

Currently, due to the rarity of clinical cases of HOCF caused by pAVMs, no consensus exists on a standardized treatment strategy. This study aims to retrospectively analyze the effectiveness of coil embolization combined with sclerotherapy in treating patients with Schobinger Stage IV.

## Materials and methods

### Patients

This study was approved by the Clinical Research Ethics Committee of China-Japan Friendship Hospital (2024-KY-246-1), and informed consent was obtained from all patients prior to data collection. In this retrospective study, all patients with HOCF caused by pAVMs who underwent embolo/sclerotherapy between May 2017 and December 2024 were included. The average age of the cohort was 42 years (range: 13–65 years), with all patients primarily receiving coil embolization combined with sclerosing agents.

Inclusion criteria were: 1. echocardiographic evidence of cardiac failure; 2. symptoms, signs, angiography, and physical examination consistent with Schobinger Stage IV; 3. lesions located in the head, neck, or limbs. Exclusion criteria included patients with congenital heart disease (right-to-left-shunt), or contraindications to polidocanol or anhydrous ethanol.

All 12 patients experienced limb edema, pain, and exertional dyspnea (100%). Echocardiography revealed varying degrees of cardiac enlargement in all 12 patients, 8 of whom had atrioventricular valve regurgitation. According to the New York Heart Association (NYHA) classification of heart failure, 6 patients were classified as NYHA IV. Other indications included hyperpigmentation (*n* = 5, 41.7%), vascular murmurs (*n* = 4, 33.3%), pulsations (*n* = 3, 25%), and ulcerations (*n* = 2, 16.7%). All patients presented with some degree of psychosocial burden at the time of presentation.

Among the 12 patients, 4 (33.3%) had undergone unsuccessful treatments at other hospitals before admission to our institution. Of these 4 patients, 1 (8.3%) had undergone stent placement, 1 (8.3%) had undergone arterial ligation, sclerotherapy, stent placement, radiofrequency ablation, and venous ligation, and 1 (8.3%) had undergone unsuccessful sclerotherapy. All 12 patients reported the presence of pAVMs at birth, and 1 (8.3%) noted recognizable expansion of the lesion during pregnancy. Baseline demographic data are presented in Table 1.

**Table 1:** Baseline demographic characteristics.

Patient no.	Age, years	Sex	Indication	NYHA classification	Previous treatment
1	65	Male	Swelling, hyperpigmentation, edema, vascular murmur	IV	Stent placement
2	38	Female	Swelling, pain, pulsation, vascular murmur	III	Feeding artery embolization, sclerotherapy
3	34	Female	Swelling, pain, hyperpigmentation	IV	Nil
4	51	Male	Swelling, pain	IV	Nil
5	64	Female	Swelling, pain, hyperpigmentation, ulceration	IV	Stent placement, radiofrequency, ablation, sclerotherapy, intravenous stitches
6	38	Male	Swelling, pain, scars, warmth, malformation	IV	Nil
7	41	Male	Swelling, hyperpigmentation, ulceration	IV	Sclerotherapy
8	27	Female	Swelling, pain, hyperpigmentation	III	Nil
9	13	Male	Swelling, pain, pulsation, vascular murmur	III	Nil
10	46	Male	Swelling, pain, vascular murmur	III	Electrochemotherapy
11	32	Male	Swelling, pain	III	Nil
12	57	Female	Swelling, pain, pulsation	III	Nil

The treatment strategy was determined through a multidisciplinary approach. The decision regarding the proposed treatment method was made following a collaborative discussion among interventional adiologists, anesthesiologists, cardiologists, respiratory specialists, and vascular surgeons.

### Technique

All procedures were performed under general anesthesia. Continuous pulmonary artery monitoring was performed if the expected total ethanol dosage exceeded 0.5 ml/kg (10).

Baseline angiography was conducted to assess the extent and hemodynamic characteristics of AVMs. The angiographic findings determined the appropriate treatment approach. The angiographic classification was based on the consensus of two interventional radiologists, in accordance with the Yakes classification system (11).

**Figure 1:**
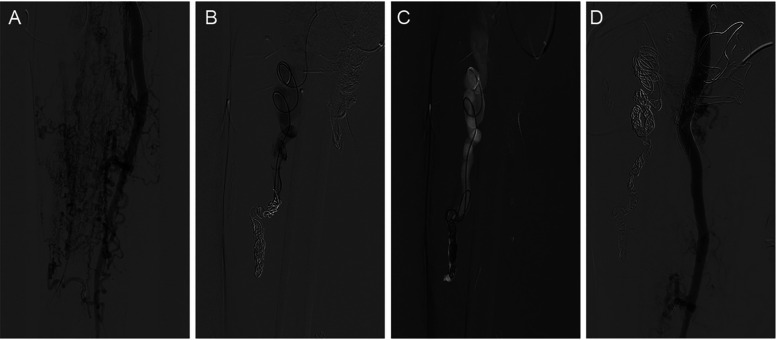
Case1 with the complaint of progressive dyspnea for 2year. A HOCF patient with right lower extremity arteriovenous malformations. **(A)** Preoperative baseline angiography shows Yakes IIIB type. **(B)** A total of 16 interlock detachable coils (IDCs) and 22 coils were used. **(C)** A total of 20 ml of 3% BPF and 5 ml of ethanol were injected. **(D)** Follow-up angiography via the right common iliac artery showed a devascularization degree of 60%.

The markedly dilated dominant drainage vein (RDDOV) was identified on the angiogram (Figure 1A). A guidewire and catheter system (Cordis, Florida, USA) were introduced through the needle and advanced into the dilated venous sac. The catheter tip was positioned as close as possible to the outlet of the RDDOV. Coils (Cook, Bloomington, IN, USA) and interlock detachable coils (IDCs) (Boston Scientific, Marlborough, Massachusetts) were deployed to embolize the RDDOV (Figure 1B). After the confirmation of slow flow in the AVM lesions, the sclerosing agent was initiated through a transvenous approach (Figure 1C). If venous embolization failed, a direct puncture approach was considered as an alternative. Postoperative angiography demonstrated the extent of devascularization achieved (Figure 1D). The technique for treating pAVMs with HOCF is summarized in Figure 1.

During the interventional embolization procedure, the injection volume and rate of the sclerosing agents were carefully controlled to ensure adequate filling of the nidus. Careful attention was given to avoiding over-injection of the sclerosing agent, as over-injection could lead to reflux, ectopic embolism and severe complications.

### Follow-up

Follow-up was completed in the outpatient clinic at 3-month intervals—specifically at 3, 6, and 12 months—including physical examinations and echocardiography. If symptoms were not relieved or recurred during the follow-up period, additional treatment was recommended.

### Statistical analysis

Statistical analysis was conducted using SPSS software version 26.0. Continuous variables were expressed as mean ± standard deviation (SD). Changes in vascular and venous pressure, as well as echocardiographic indices between baseline and the final treatment session, were assessed using a paired *t*-test. Pearson correlation analysis was conducted to examine the relationship between a reduction in venous drainage pressure and LVEF. A two-sided *p*-value of <0.05 was regarded as statistically significant.

## Results

### Degree of devascularization and improvement in symptoms

Super-selective angiography revealed that AVMs were located in the left lower limb (*n* = 7, 58.3%), right lower limb (*n* = 4, 33.3%), and right upper limb (*n* = 1, 8.3%) in the 12 patients. According to the Yakes classification, all 12 patients were classified as Type III B (*n* = 4, 33.3%), Type II B (*n* = 4, 33.3%), Type IV (*n* = 2, 18.3%), III A (*n* = 1, 8.3%), and Type II A (*n* = 1, 8.3%).

A total of 24 embolo/sclerotherapy sessions were performed (mean 2; range 1–3). The results demonstrated substantial efficacy, with 5 patients (41.7%) achieving >90% devascularization, 4 patients (33.3%) achieving 80%, and 3 (25.0%) patients achieving >60%, as shown in Table 2.

**Table 2:** Intraoperative details.

Patient no.	Location	Yakes type	No. of sessions	Number of coils		Volume of polidocanol (ml)	Volume of ethanol (ml)
				Interlock	Cook		
1	RLE	IIIA	2	16	19	36	9
2	LLE	IIB	2	28	154	24	5
3	RLE	IIIB	2	3	9	28	5
4	RUE	IIIB	2	4	5	32	7
5	RLE	IIIB	1	7	25	17	5
6	LLE	IIB	3	8	20	47	7
7	LLE	IIIB	2	5	28	35	9
8	LLE	IIB	2	11	27	35	7
9	LLE	IV	2	5	3	24	7
10	LLE	IIB	2	5	23	30	9
11	RLE	IV	2	4	13	27	5
12	LLE	IIA	2	5	11	20	5

LLE, left lower extremity; RLE, right lower extremity; RUE, right upper extremity.

All the 12 patients initially classified as Schobinger stage IV were downgraded to stage I (*n* = 10, 83.3%) and stage II (*n* = 2, 16.7%) following the procedure. Dyspnea was the primary reason for seeking intervention in all 12 patients. Postoperatively, 2 (16.7%) had complete relief, and 10 (83.3%) experienced major improvement in dyspnea. No significant complications were encountered during the hospitalization of the 12 patients as shown in Table 3.

**Table 3:** Clinical outcomes and embolization degree of patients undergoing endovascular treatment.

Patient no.	Complications	Follow-up period, months	Degree of devascularization (%)	Schobinger classification post embolization	Outcome
1	None	12	60%	I	Major improvement
2	None	12	90%	I	Complete relief
3	None	6	80%	I	Major improvement
4	None	12	80%	II	Major improvement
5	None	3	80%	II	Minor improvement
6	None	6	95%	I	Major improvement
7	None	12	60%	I	Major improvement
8	None	6	95%	I	Complete relief
9	None	12	60%	I	Major improvement
10	None	12	95%	I	Major improvement
11	None	6	80%	I	Major improvement
12	None	12	98%	I	Major improvement

Postoperatively, the patients' previously high pressure in venous drainage (*P* = 0.049) due to nidus decreased significantly, but not in vascular drainage (*P* = 0.395), Table 4.

**Table 4:** Comparison between pre- and post-operative pressure in arterial feeders and venous drainage of nidus.

Variable	Mean of difference (95% CI)	*P*
Pressure changes in arterial feeders (mmHg)	−2.67 (−9.29, 3.96)	.395
Pressure changes in venous drainage (mmHg)	−16.75 (−33.46, −0.04)	**.** **049**

CI, confidence interval.

Bold values indicate statistical significance.

### Echocardiography result

Echocardiographic examination revealed that, postoperative regurgitation was reduced in all 8 (66.7%) patients who had valvular regurgitation as shown in Table 5. The preoperative dilation of left ventricular end-diastolic dimension (LVDd) (*P* < 0.001), left ventricular end-systolic dimension (LVDs) (*P* = 0.016), right atrial diameter (RAD) (*P* = 0.002), and right ventricular basal diameter (RVD1) (*P* = 0.002) all decreased with statistical significance, as well as the elevated pulmonary artery systolic pressure (PASP) (*P* = 0.034). Also, the LVEF improved after surgery (*P* < 0.01) as shown in Table 6.

**Table 5:** Pre- and post-operative valvular regurgitation of all 12 patients.

Patient No.	Preoperative		Postoperative	
	MR	TR	MR	TR
1	Moderate	Severe	Mild-moderate	Moderate
2	None	None	None	None
3	Mild-moderate	Moderate	Mild-moderate	Moderate
4	None	None	None	None
5	Mild-moderate	Moderate	Mild-moderate	Mild-moderate
6	Mild-moderate	Severe	Mild-moderate	Moderate
7	None	None	None	None
8	Mild-moderate	Moderate	None	Moderate
9	None	None	None	None
10	Moderate	Moderate	Mild-moderate	Moderate
11	None	Moderate	None	None
12	None	Moderate	None	Mild-moderate

MR, mitral regurgitation; TR, tricuspid regurgitation.

**Table 6:** Comparison of echocardiographic data before and after surgery.

Variable	Mean ± SD	*P*
LVDd, mm	6.08 ± 2.96	**.** **001**
LVDs, mm	6.17 ± 4.79	**.** **016**
RAD, mm	5.33 ± 2.89	**.** **002**
RVD1, mm	6.42 ± 3.47	**.** **002**
LVEF, %	7.58 ± 7.17	**.** **004**
PASP, mmHg	3.58 ± 3.27	**.** **034**

Data are presented as mean ± standard deviation. Bold values indicate statistical significance.

LVDd, left ventricular end-diastolic dimension; LVDs, left ventricular end-systolic dimension; RAD, right atrial diameter; RVD1, right ventricular basal diameter; LVEF, left ventricular ejection fractions; PASP, pulmonary artery systolic pressure.

### Correlation between decreased pressure in venous drainage and LEVF in patients with HOCF caused by pAVMs after embolo/sclerotherapy

**Figure 2:**
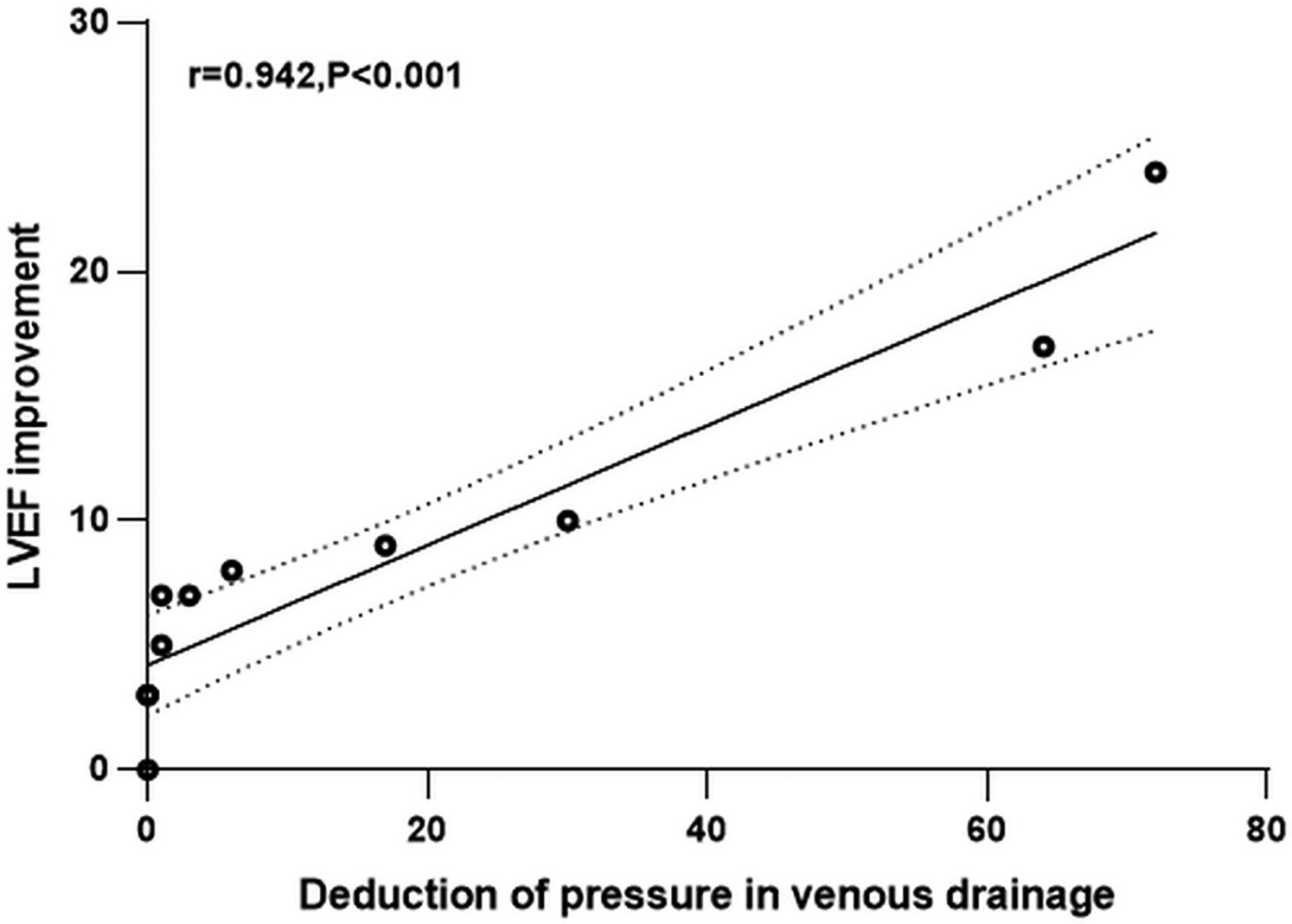
Scatter plots of correlation between changes in venous drainage pressure and LVEF improvement of patients with HOCF caused by pAVMs after embolo/sclerotherapy.

Excluding one case of elevated venous drainage pressure following surgery, further analysis of the linear relationships between reductions in venous drainage pressure, devascularization, and the improvement of LVEF in 11 patients with HOCF caused by pAVMs as shown in Table 7, indicated that LVEF improvement (*P* < 0.001) was significantly positively correlated with decrease in venous drainage pressure of nidus (*r* = 0.923) through surgery, and the scatter plots of correlation were plotted in Figure 2.

**Table 7:** The correlation between pressure in venous drainage, devascularization and left ventricular ejection fraction in patients before and after embolo/sclerotherapy.

Variable	LVEF improvement (%)	
	*r*	*P*
Pressure changes in venous drainage (mmHg)	0.942	**<0** **.** **001**
Devascularization (%)	0.005	0.989

Pearson correlation analyses were used. Bold values indicate statistical significance.

## Discussion

AVMs are the second leading cause of HOCF (12). HOCF resulting from pAVMs remains a challenging clinical issue. Severe pAVMs may result in systemic arterial hypotension and neurohormonal activation. However, the use of conventional heart failure medications may reduce systemic vascular resistance, potentially exacerbating the condition (13). Historically, treatment options for AVMs included resection and ligation, which, while effective, were associated with significant complications, such as increased intraoperative bleeding risk and limited impact on recurrence rates (14).

The advancement of endovascular techniques and novel embolic agents has made embolization and sclerotherapy the primary treatments for AVMs. These methods significantly improve outcomes by reducing the flow rate in major draining veins and addressing the underlying causes of high-output states (15–17). The primary treatment goal for pAVMs with HOCF is to eliminate abnormal pathways and reduce cardiac volume overload, thereby improving patients' quality of life.

Endovascular embolization for pAVMs includes transarterial, transvenous, and direct puncture approaches. Compared with the transarterial route, the transvenous approach may achieve curative embolization of pAVMs more effectively (18). Studies have shown that transvenous pAVM embolization is associated with higher complete occlusion rates and relatively lower complication and recurrence rates (19). Although veins are generally thinner than arteries, the arterialized venous walls found in AVMs are thicker (20). Additionally, advances in microcatheter and microwire technologies have improved the accuracy and safety of transvenous catheterization in recent years. Furthermore, the direct puncture approach enables more precise delivery of embolic agents to the nidus.

In this study, a treatment strategy combining transvenous embolization and direct puncture approaches was chosen for the 12 patients. To mitigate potential complications from percutaneous puncture, such as hematoma or embolic agent leakage at the puncture site, manual compression time at the puncture site was extended post-procedure. Consequently, no complications were observed in this study.

In embolo/sclerotherapy, the use of coils offers advantages in limiting the spread of embolic agents, reducing complications, and minimizing intraoperative bleeding (21). This approach helps control the reflux of embolization materials, facilitating complete occlusion of fistulous areas and potential abnormal shunts (16, 22). The choice of coil length and stiffness depends on the location and morphology of the malformation. High coil density within the vessel is essential for effectively reducing flow velocity (23, 24). Surgeons must measure and operate precisely to prevent complications, such as ectopic embolization. Additionally, the use of conventional coils in combination with IDC coils improves localization and release accuracy, allowing for easier adjustments (25). After transvenous embolization, some patients received ethanol injections into the feeding artery of AVMs, followed by transarterial embolization. This approach significantly improved the efficacy of transvenous embolization and reduced recurrence rates when compared to pure arterial embolization.

In the previous study, we evaluated the safety and efficacy of BPF as a sclerosing agent (26). Both polidocanol and bleomycin are widely used as clinical sclerosants. Polidocanol exhibits concentration-dependent efficacy in occluding blood vessels, while bleomycin promotes vascular endothelial damage and fibrosis (27, 28). To enhance drug contact with the vasculature and reduce washout rates in the lesion area, BPF is typically prepared as foam for clinical use. Our previous study confirmed that BPF achieves greater lesion volume reduction in AVM treatment than polidocanol foam alone (29). Anhydrous ethanol, while highly effective in symptom improvement or resolution in AVM patients (30), is associated with significant toxicity (31), particularly cardiotoxicity, such as arrhythmias and right heart failure (32, 33). Given that all patients in this study presented with HOCF, a combination of anhydrous ethanol and the milder BPF was chosen to balance efficacy and safety (8, 34, 35).

HOCF, although rare, represents a severe complication in pAVM patients. Our approach aimed to improve cardiac function through devascularization. While previous studies primarily assessed cardiac improvement using NYHA functional class and clinical indicators, this study also analyzed echocardiographic results. Comparisons of pre- and post-operative echocardiograms revealed significant cardiac remodeling, with statistically significant improvements in LVDd, LVEF, and PASP, among other parameters. These findings provide strong evidence of improvement in cardiac function. While some studies classify this type of cardiac failure as preserved ejection fraction heart failure, delayed diagnosis and treatment may have resulted in reduced ejection fraction.

This study demonstrated a linear relationship between the reduction in venous pressure in the nidus drainage and the improvement of LEVF in patients with HOCF caused by pAVMs through embolo/sclerotherapy. Previously, postoperative devascularisation was considered a factor contributing to improvements in cardiac failure associated with pAVMs; however, our study found no statistical significance related to this.

All 12 patients in this study experienced significant psychosocial stress due to their malformations. The psychological burden was exacerbated by the chronicity and progression of the condition, as many patients had lived with their malformations for extended periods before seeking treatment. All patients noted significant improvement in the appearance of the affected limb and expressed satisfaction with the cosmetic outcomes post-surgery. These findings underscore the importance of addressing both physical and psychological aspects of pAVMs to enhance patients' quality of life.

This study has limitations. High-flow AVMs require timely and early treatment, but late presentation with severe heart failure symptoms and the high cost of treatment presented challenges. The small sample size and loss to follow-up due to prolonged staged treatment limited the study. As a retrospective study, future research should incorporate prospective designs with larger patient cohorts for more accurate evaluation and enhanced follow-up protocols.

## Data Availability

The original contributions presented in the study are included in the article/Supplementary Material, further inquiries can be directed to the corresponding authors.
